# Changes in ecological conditions may influence intraguild competition: inferring interaction patterns of snow leopard with co-predators

**DOI:** 10.7717/peerj.14277

**Published:** 2022-10-25

**Authors:** Ranjana Pal, Anshu Panwar, Surendra P. Goyal, Sambandam Sathyakumar

**Affiliations:** Department of Endangered Species Management, Wildlife Institute of India, Dehradun, Uttarakhand, India

**Keywords:** Common leopard, Woolly wolf, Occupancy, Interspecific interactions, Temporal overlap, Scat analysis

## Abstract

**Background:**

Large-scale changes in habitat conditions due to human modifications and climate change require management practices to consider how species communities can alter amidst these changes. Understanding species interactions across the gradient of space, anthropogenic pressure, and season provide the opportunity to anticipate possible dynamics in the changing scenarios. We studied the interspecific interactions of carnivore species in a high-altitude ecosystem over seasonal (summer and winter) and resource gradients (livestock grazing) to assess the impact of changing abiotic and biotic settings on coexistence.

**Methods:**

The study was conducted in the Upper Bhagirathi basin, Western Himalaya, India. We analyzed around 4 years of camera trap monitoring data to understand seasonal spatial and temporal interactions of the snow leopard with common leopard and woolly wolf were assessed in the greater and trans-Himalayan habitats, respectively. We used two species occupancy models to assess spatial interactions, and circadian activity patterns were used to assess seasonal temporal overlap amongst carnivores. In addition, we examined scats to understand the commonalities in prey selection.

**Results:**

The result showed that although snow leopard and wolves depend on the same limited prey species and show high temporal overlap, habitat heterogeneity and differential habitat use facilitate co-occurrence between these two predators. Snow leopard and common leopard were spatially independent in the summer. Conversely, the common leopard negatively influences the space use of snow leopard in the winter. Limited prey resources (lack of livestock), restricted space (due to snow cover), and similar activity patterns in winter might result in strong competition, causing these species to avoid each other on a spatial scale. The study showed that in addition to species traits and size, ecological settings also play a significant role in deciding the intensity of competition between large carnivores. Climate change and habitat shifts are predicted to increase the spatial overlap between snow leopard and co-predators in the future. In such scenarios, wolves and snow leopards may coexist in a topographically diverse environment, provided sufficient prey are available. However, shifts in tree line might lead to severe competition between common leopards and snow leopards, which could be detrimental to the latter. Further monitoring of resource use across abiotic and biotic environments may improve our understanding of how changing ecological conditions can affect resource partitioning between snow leopards and predators.

## Introduction

Carnivore species exhibit strong antagonistic interactions, and their distribution is often regulated by interspecies competition (Donadio & Buskirk, 2006; Fisher et al., 2013; Santulli et al., 2014). Partitioning across multidimensional niches (*e.g*., space, activity, and diet) reduces exploitative competition and the likelihood of interspecies encounters (Karanth & Sunquist, 1995; Dröge et al., 2017). If unable to do so, increased competition among species may lead to the exclusion of weaker competitors, possibly leading to local extinction (Pimm, 1991; Rettelbach et al., 2013) with a cascading effect on ecosystem stability (Borrvall, Ebenman & Tomas Jonsson, 2000). Increasing evidence suggests that anthropogenic influences such as habitat fragmentation and habitat alterations intensify carnivore competition (Lewis et al., 2015; Cruz et al., 2018; Smith et al., 2018). For example, a study conducted by Parsons et al. (2019) on carnivore interactions across the urban gradient found that fragmentation leads to higher levels of spatial interaction. A similar analysis of niche partitioning along the gradient of human disturbance in North America featuring sympatric apex and mesocarnivores revealed that humans alter resource niches, influencing competitive interactions (Manlick & Pauli, 2020). Future climate change can further exacerbate the negative interspecies interactions (Rubidge et al., 2011; Ockendon et al., 2014) and can have a detrimental impact on species status. For example, Cunningham, Rissler & Apodaca (2009) showed that under stressful environmental conditions salamanders lost biomass influencing the competitive abilities of adults. Similarly, a study across the climate gradient showed that martens and fishers were allopatric in the wetter region but sympatric in the drier environment (Zielinski, Tucker & Rennie, 2017).

A variety of factors determine the level of competition between species. Body size appears to be the most crucial factor in interspecies killing probability in carnivores (Donadio & Buskirk, 2006). The perceived risk of exploitative competition is likely to incur costs, such as lower activity, decreased foraging rate or efficiency, or greater use of refugia (Pauli et al., 2022). Interactions between species with similar dietary niches exhibit high levels of interference competition (Pauli et al., 2022). Antagonistic interactions are partially pronounced in species with similar traits, evolutionary relatedness, or species belonging to the same family (Donadio & Buskirk, 2006). Additionally, owing to huge variations in landscape features and resource abundance, species interactions and the associated behaviour may also vary seasonally (Torretta et al., 2016; Finnegan et al., 2021).

High-altitude ecosystems of Asia are fragile, understudied, and threatened by habitat loss, prey depletion, poaching, and retaliatory killing (Snow Leopard Network, 2014; McCarthy et al., 2017). Human population growth and economic development are escalating the extent to which these activities result in habitat fragmentation and degradation (Li et al., 2016). Additionally, global climate change is expected to exacerbate the problems that high-altitude species endure by decreasing the total available habitat and increasing the distance between appropriate habitat patches (Diaz, Grosjean & Graumlich, 2003; Forrest et al., 2012). Snow leopard (*Panthera uncia*), an apex predator endemic to Asia’s high ranges, mostly inhabits the alpine zone at 3,000–5,000 m, a climate-sensitive area (Farrington & Li, 2016). Climate-based habitat models for the snow leopard range suggest a shift in the habitat, particularly in the greater Himalayan region (Forrest et al., 2012; Lovari et al., 2013a; Aryal et al., 2016). The future survival of snow leopard in this region largely depends upon the ecological plasticity and adaptive potential to survive in the changes brought by global warming (Li et al., 2016). One of the major challenges for snow leopards is expected to be direct or indirect competition for resources with other carnivores, such as wolves and common leopard. The competition between these species, however, is still poorly understood.

To address these knowledge gaps, we sought to understand the mechanisms that facilitate coexistence between snow leopards and co-occurring predators. Snow leopard habitat in the Himalayan region overlaps with common leopard in its southern distribution range. The diversity and abundance of prey species are generally low in high-altitude landscapes; hence, similar-sized carnivores are likely to compete for food (Karanth et al., 2017; Santos et al., 2019; Jumabay-Uulu et al., 2014). Because both felids species have comparable body size and morphology (with the common leopard being the larger species), competitive interactions between both leopards are expected in areas of geographic overlap (Lovari et al., 2013a). In much of its range, including its northern range in the Himalayas, the distribution of the snow leopard coincides with that of the woolly wolf. Due to its size, social living, and generalist behaviour, the woolly wolf is expected to outcompete the snow leopard (Lovari & Mishra, 2016; Lyngdoh, Habib & Shrotriya, 2020). While competition for prey in snow leopard and wolves has been well studied (Jumabay-Uulu et al., 2014; Wang et al., 2014; Chetri, Odden & Wegge, 2017), spatial segregation has not received much attention.

We assessed seasonal co-existence patterns of snow leopard with co-occurring predators at spatial and temporal scales in the Upper Bhagirathi landscape. The landscape is a tourist hotspot and grazing grounds for livestock in summer. Grazing does not occur during winters (November to April), and human presence is relatively low (Pal et al., 2021a; Pal et al., 2022a; Pal et al., 2022b). For the study, we focused on understanding interaction under two scenarios: summer (warmer, snow-free, and livestock grazing season) and winter (cold, snow cover, and nongrazing season) months. Summer indicated snow-free conditions with more area availability and additional food resource as livestock. Conversely, space use is limited in winter due to thick snow cover and reduced availability of prey because there are no livestock on the landscape. It is expected that most agonistic encounters between predator species occur in seasonal environments when food is scarce (Palomares & Caro, 1999). In addition to space and temporal patterns, we investigated the dietary similarity or dissimilarity of snow leopard with other two predators in their region of co-occurrence. To investigate the influence of habitat conditions and resource availability on carnivore interactions, we developed the following hypothesis: (i) Owing to large size (~35 kg, Hennelly et al., 2017), generalist diet, and pack living habits, the wolf will negatively influence the space use of snow leopard (body weight: female 25–40 kg, male 45–75 kg; Nowark, 1991; Jackson, 1996). Similarly, (ii) common leopard, (body weight: female 28–60 kg, male 37–80 kg; Nowark, 1991) owing to its larger size, might negatively influence the space use of snow leopard. (iii) Competition may intensify between species in winters due to restricted space and lack of food from human sources (livestock). The study provides crucial insights into the less studied nature of the snow leopard and its interlinked relationship with other co-occurring carnivores. Understanding these processes is critical to delineating important areas for species conservation (Linnell & Strand, 2000).

## Materials and Methods

### Study area

**Figure 1:**
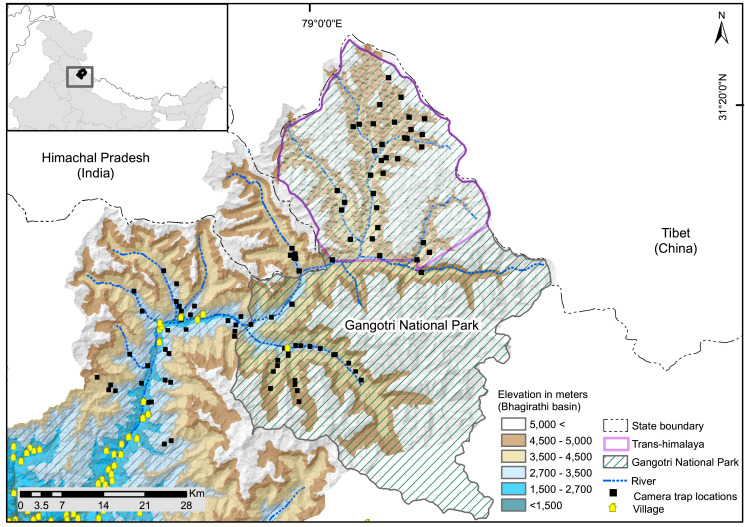
Map of study area showing camera trap locations in greater Himalayan region and trans-Himalayan region in the Upper Bhagirathi basin, Western Himalaya, India.

The study was conducted in the upper catchment of the Bhagirathi River (2,700 to 3,000 m), a tributary of the Ganga also known as the Gangotri landscape (~4,600 km^2^, 2,700 to 5,000 m). It is located in the northwestern part of the state of Uttarakhand in the western Himalayan region of India (Fig. 1). The study area comprises of Trans-Himalayan (Nelang valley) and the greater Himalayan region (Gangotri valley) of Gangotri National Park, and the surrounding greater Himalayan region (Fig. 1). Monitoring data based on weather station situated at 3,700 m showed that temperatures range between −2.3 and 11 °C (2001–2009, Orr et al., 2019). The weather station has recorded a mean annual winter snowfall of ~546 mm (Bhambri et al., 2011). The area between 2,700–3,000 m altitude is represented by temperate vegetation consisting of Oak (*Quercus* spp), rhododendron, Himalayan cypress (*Cupressus torulosa*) and deodar (*Cedrus deodara*). The subalpine zone (3,000–3,600 m) is formed by *Betula utilis, Rhododendron* sp., *Pinus wallichiana*, *Taxus wallichiana* with intermixed broad-leaved trees like Kharsu oak (*Quercus semecarpifolia*). Alpine area of Greater Himalaya consist of tall, forbes, mixed herbaceous formations, *Danthonia* grasslands, *Kobresia* sedge, often mixed with cushioned species and junipers or rhododendron scrubs (Pusalkar & Singh, 2012). Trans-Himalayan valleys (Nelang valley) consist of steppe scrub vegetation dominated by *Artemisia* spp., *Astragalus candolleanus*, *Caragana* sp., *Hyssopus officinalis*, *Oxytropis* spp (Pal et al., 2022b). In the Trans-Himalayan region, snow leopard and woolly wolf are the large predators, and potential wild prey include bharal (*Pseudois nayaur*), Tibetan woolly hare (*Lepus oiostolus*), and Himalayan marmot (*Marmota himalayana*) (Pal et al., 2022b). Tibetan argali (*Ovis ammon*) is rare in the region (Chandola, 2008; Pal et al., 2021a, 2022b). In the greater Himalayan region, snow leopard co-occurs with carnivores species such as Himalayan brown bear (*Ursus arctos isabellinus*) and common leopard (*Panthera pardus*) and potential prey species are: bharal, musk deer (*Moschus* sp.), Himalayan tahr (*Hemitragus jemlahicus*), goral (*Naemorhedus goral*), serow (*Capricornis thar*) and sambar (*Rusa unicolor)*.

There are nine major villages in the study area (Fig. 1), all of which are located below the elevation of 3,000 m. Landscape has one protected area: Gangotri National Park. In summer (June to September) livestock are grazed, except in two valleys within the protected area. Tourism, mountaineering, and pilgrimage attract many visitors between April and November. The Gangotri National Park remains closed for visitors in winter (November to April). The northern boundary of the study area forms the international border with the Tibet region of China. Patrolling camps and small settlements of the Indo-Tibetan Border Police and other security agencies are present. Analysis of seasonal anthropogenic pressure in the UBB showed a low presence of humans and associated activities in winter compared to summer (Pal et al., 2021a). Livestock grazing, developmental and tourism activities, poaching, and advancement of military infrastructure are potential threats to species inside and outside protected areas in the landscape (Chandola, 2008; Bhardwaj, Uniyal & Sanyal, 2010). In addition, studies have observed notable warming trends in the area that may alter resource availability (Alexander et al., 2016), threatening the long-term existence of high altitude species in the region. Glacier retreat has been observed in the Gangotri and Dokriani glaciers (Kumar et al., 2008; Dobhal, Gergan & Thayyen, 2004). The increase in taxa such as *Juniperus, Betula, Salix*, and *Pinus* in the high-altitude areas of Gangotri indicates that temperature and precipitation are increasing in this region (Kar et al., 2002).

### Field surveys

**Figure 2:**
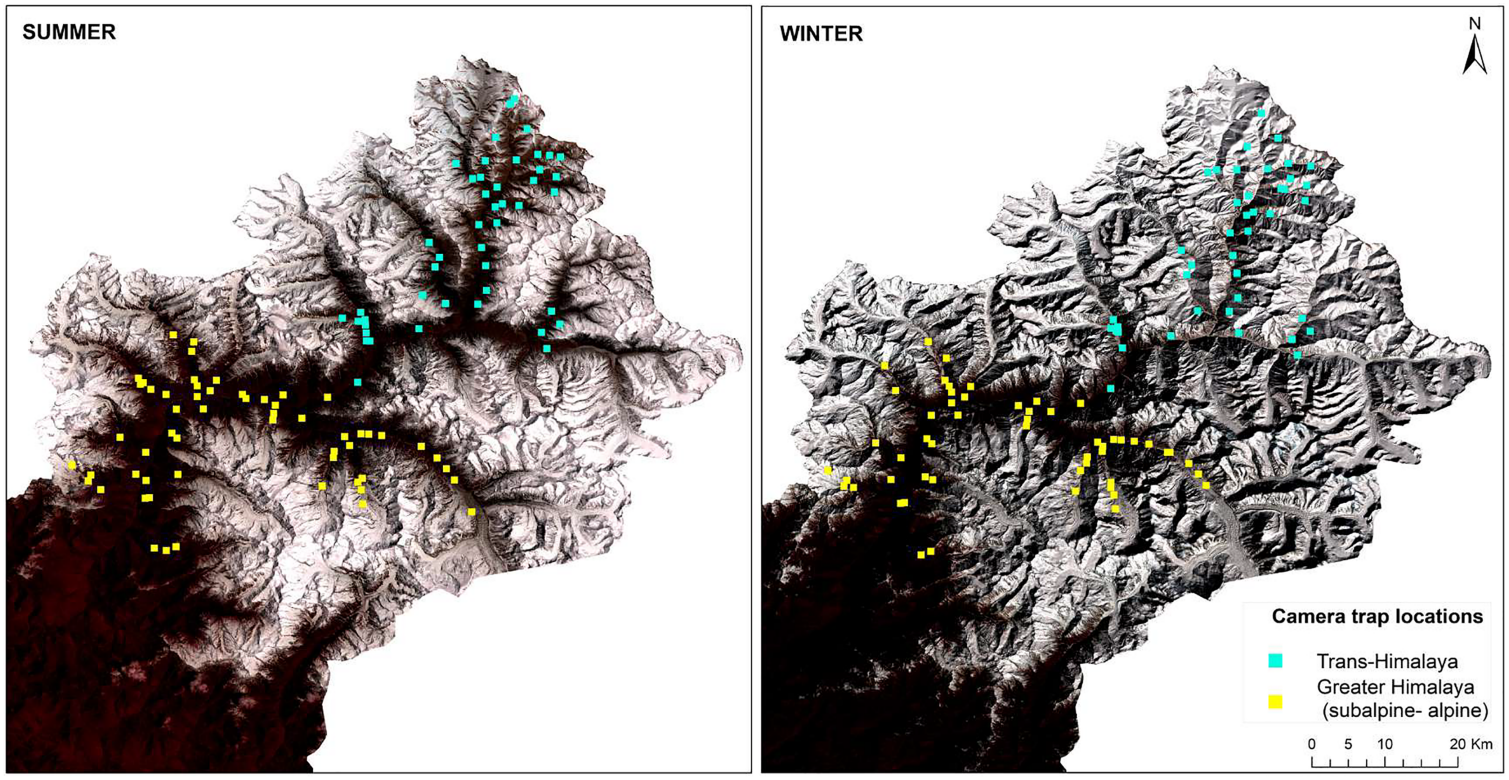
Standard false colour composite map of Upper Bhagirathi basin, Western Himalaya, India showing the sampling locations and habitat conditions in summer and winter.

This study was conducted with the permission and support of the Uttarakhand Forest Department (Letter No. 836/5-6, 702/5-6 and 117/5-6). Camera trapping was carried out using 3 × 3 km grids in different high-altitude valleys of the Upper Bhagirathi basin. Sampling was carried out between October 2015 and March 2019, focusing on two seasons: summer (May to September) and winter (November to March) (Fig. 2). The length of the sessions was chosen to maintain consistency in terms of anthropogenic disturbance and season. Human activities such as grazing, construction, and paramilitary presence increases in summer (May to September). In contrast, livestock grazing is prohibited and the area is covered up in snow from November to March (Pal et al., 2022a, 2022b). Camera traps (C1 Cuddeback, De Pere, WI, USA) were used to assess the seasonal habitat use of species along a 2,500–5,000 m elevation gradient. Camera traps were deployed in locations likely to be used by mammals based on signs such as scats and pugmark/pellets, at a height of approximately 30–45 cm above the ground. Due to logistical constraints, each camera site could monitored only once per season. Data for summer 2017 (66 sites) and winter 2017–2018 (62 sites) for subalpine and alpine habitats (2,700–3,500 m) were used to analyze space use overlap between snow leopard and common leopard. Data for summer 2018 (47 sites) and winter 2018–2019 (43 sites) for the Trans-Himalayan region (3,200–5,000 m) were used to analyze space use overlap between snow leopard and woolly wolf.

### Data analysis

#### Spatial co-occurrence

Single-season two-species occupancy in a Bayesian framework was used to determine the extent to which two sympatric predators simultaneously inhabit an area and whether co-occurring carnivore species (Trans-Himalaya: woolly wolf; greater Himalaya: common leopard) affect the site occupancy and detection likelihood of snow leopard. These conditional co-occurrence modeling methods (1) account for imperfect detection of all target species; (2) estimate the occupancy of multiple species; and (3) determine whether the presence of one species influences the occupancy or detection of the other (MacKenzie, Bailey & Nichols James, 2004). The occurrence of smaller species (here, snow leopard) is assumed to be dependent on the presence of larger species (here, woolly wolf and common leopard), but the presence of the larger species is independent of the other species (Farris et al., 2014). The scale (3 × 3 km) of the present study was much smaller than the home range size of snow leopard (adult male: 207 km^2^ ± 63 SD; adult female: 124 km^2^ ± 41 SD, Johansson et al., 2016) and individuals may not be available for capture on consecutive sampling days. Collapsing survey periods helps reduce overdispersion and increase temporal independence of detections (Penjor et al., 2022). Therefore, to avoid zero inflation in the data (*i.e*., too many non-detections) and increase detection frequency, multiple days were pooled as one occasion, an approach commonly used in large carnivore occupancy analysis (MacKenzie & Royle, 2005; Tobler et al., 2015). Matrices were developed by pooling 6 days of survey as one occasion in columns and rows consisting of camera locations. For each observation, ‘1’, ‘0’, or ‘-’ was designated for each observation, where ‘1’ indicated one or multiple occurrences within the particular occasion, ‘0’ indicated no record and ‘-’ no sampling during those occasions. Multiple photo captures on one occasion were considered as a single detection. The co-occurrence model includes a “species interaction factor” (SIF) parameter, a measure of interaction to determine whether two target species co-occur independently (MacKenzie, Bailey & Nichols James, 2004). A SIF value less than one indicates that the species is less likely than expected to co-occur with the dominant species (indicating avoidance), whereas a SIF value greater than one indicates that the species is more likely than expected to co-occur with the dominant species (indicating attraction). The SIF value equal to one indicates that the two species occur independently. Analysis was done in R (R Core Team, 2020) using the package R2Jags (Plummer, 2011). Posterior distributions were estimated by generating three chains of 1,000 iterations after a burn-in of 10,000 iterations (Davis et al., 2018). For all parameters, uninformative priors were drawn from a uniform distribution of 0 to 1. Model convergence was assessed based on visual inspection of chain trace plots and R^2^ values, where a value closer to 1.0 indicates a more plausible convergence (Davis et al., 2018). No covariates were used for this analysis as models with covariates failed to converge.

#### Activity pattern

To understand activity patterns, a larger dataset of camera trap monitoring from October 2015–March 2019 was used. The activity was assessed based on the date and time information on the camera trap photographs, assuming that the number of photographs taken correlated with carnivore activity levels (Kawanishi, 2002). Repeated captures of the same individuals on the same occasion were considered as one capture. All the captures of species in different years were pooled according to the winter and summer seasons to determine the general activity pattern of the species. The time of capture of species was first converted into radians to account for the circular distribution of the time of the day (Meredith & Ridout, 2014). Following that, a non-parametric kernel density function was used to estimate the daily activity patterns (Ridout & Linkie, 2009). The coefficient of overlap ( $\Delta$) was used to calculate the activity overlap for the species pairs for each season. The value ranges from 0 to 1 and indicates the degree of overlap between two kernel density estimates (*i.e*., the daily activity patterns of two species compared) (Ridout & Linkie, 2009). Due to the small sample size, we used the overlap coefficient ‘ $\Delta_1$’ as recommended by Ridout & Linkie (2009). The “overlap” package (Meredith & Ridout, 2014) in R was used for the temporal overlap analyses.

#### Diet

Scats encountered during camera trap surveys, once per season from June 2015 to July 2019, were opportunistically collected for food habits investigation. In the field, scat samples were identified based on their size and appearance. Since field misclassification of scats is common in the focal species (Jumabay-Uulu et al., 2014; Weiskopf, Kachel & McCarthy, 2016), these were later confirmed using mtDNA analysis. The mtDNA and diet preference analysis was done as previously described in Pal et al. (2022b). Scats were oven-dried at 40 °C until no moisture was detected in the centre of the sample (Piggott & Taylor, 2003). Post drying the samples, only the upper mucosal layer of scat was scraped and 0.2 g was taken for genetic analysis. The remainder was stored in plastic bags with silica (silica:scat = 4:1) at room temperature for food habit analysis. DNA was extracted using a QIAamp DNA stool kit (Qiagen, Hilden, Germany), following the Qiagen manual with slight modifications. Negative control was also run with the samples to check exogenous DNA contamination. For the amplification of mtDNA carnivore-specific primers 146 bp long cytochrome b gene, F 50-TATTCTTTATCTGCCT ATACATRCACG-30; R 50-AAACTGCAGCCCCTCAGAATGATATTTGTCCTCA-30 (Farrell, Roman & Sunquist, 2000); 144 bp long ATP6-DF3; 50-AACGAAAATCTATTCGCCTCT-30; DR1, 50-CCAGTATT TGTTTTGATGTTAGTTG-30 (Chaves et al., 2012); and 12S rRNA, namely, F 50-AAAAAGCTTCAAACTGGGATTAGATACCCCACTAT-30 and R 50-TGACTGCAGAGGGTGACGGGCGGTGTGT-30 (Kocher et al., 1989) were used. These primers are known to maximize efficiency even in degraded samples. DNA was amplified in 10 µL reaction mixture containing 10× PCR buffer that includes 20 mM MgCl_2_, 20 mg/ml BSA, 2 mM (each) dNTPs mix, 0.05 mmol primer, 5 U/ml Taq DNA polymerase (Thermo Fisher Scientific, Vilnius, Lithuania), and distilled water. PCR conditions were followed by initial denaturation at 95 °C for 3 min, 40 cycles of denaturation at 95 °C for 30 s, annealing at 52 °C (cytb), 55 °C (ATP6) and 60 °C (12 s rRNA) for 45 s, 72° Cfor 55, and final extension for 10 min at 72 °C. Sequencing of amplified PCR products was first performed using exonuclease-I and shrimp alkaline phosphatase (Thermo-Scientific Inc., Waltham, MA, USA) for the removal of free dNTPs and primer residues. The forward primer of each fragment was used for the BigDye version 3.1 kit cycle sequencing. Later, the fragments were washed using sodium acetate to precipitate the DNA, followed by chilled alcohol. Then, the clean desired DNA fragments were sequenced using Applied BioSystem Bio-Analyzer 3500 XL. Subsequently, the obtained sequence was examined by searching BLAST (NCBI, Bethesda, MD, USA) to identify the closest homologous similarity of the unknown sequence. DNA samples that showed the highest similarity with reference sequences (NCBI, Bethesda, MD, USA) were considered distinguishable in terms of species identification.

Scats were washed in running water through a fine 125 mm sieve to ensure that no digested material passed through the sieve. Indigestible items (hair, feathers, bones, claws, teeth, chitin remnants of insects, seeds, grasses, and other plant materials) and human-derived materials (cloth, paper, plastic, and rubber) were collected for further identification. After drying the derived hair and feather samples in a 40 °C oven for 48 h, the hair was washed and water mounted for examination under a microscope (40 or 100). For analysis, we randomly selected 20 hair samples from each scat (Jethva & Jhala, 2003). We collected wild prey reference hair samples from carcasses found while fieldwork. We used the database of the Wildlife Institute of India (Bahuguna et al., 2010) and published reference keys (Oli, 1993) for the species for which reference samples could not be collected in the field. Color, length, thickness, characteristic medullar configurations, cortex-to-medulla ratio, and cuticle pattern were used to identify prey (Reynolds & Aebischer, 1991; Mukherjee, Goyal & Chellam, 1994). Rodent species (with the exception of the Himalayan marmot) could not be identified as species and were placed in a broad category as “small prey.”

Genetic confirmation was possible only in 32, 54, and 24 for woolly wolf, snow leopard, and common leopard, respectively. Since we had a limited number of scats, we could not study the seasonal food habit and dietary overlap between species. We pooled all the scats to determine the general pattern of prey consumed by each predator. The frequency of occurrence (FO) per item as F = n/N, was used to calculate the utilization rate of different food items, where n is the number of scats with a particular species and N is the total number of scats analyzed (Karanth & Sunquist, 1995; Bashir et al., 2014). We subjected the results of the occurrence analysis to resampling using the bootstrap method. Subsamples equaling the original sample size of scats were iterated 10,000 times to generate means and bias-corrected 95% confidence intervals for the percentage frequency of prey items in scats (Mukherjee et al., 2004). When prey differs in size, utilisation indices, particularly FO, tend to underestimate the share of large prey compared to small prey (Klare, Kamler & Macdonald, 2011). Therefore, the frequency of occurrence of prey species in scats was converted to relative biomass (Karanth & Sunquist, 1995; Bashir et al., 2014), which provides the best approximation of actual food habits (Klare, Kamler & Macdonald, 2011). To estimate food intake per prey species, the following biomass calculations were used:

(i) For snow leopard: Y = 1.980 + 0.035X (Ackerman, Lindzey & Hemker, 1984)
(ii) For woolly wolf: Y = 0.439 + 0.008X, (Weaver, 1993)
(iii) For common leopard: Y = 2.171 − 1.671 exp-0.056X (Chakrabarti et al., 2016)

where Y the biomass of prey consumed to produce a scat, and X the average body weight of each prey species.

Dietary overlap among carnivore species was evaluated using the niche overlap index (O) (Pianka, 1973). This index also varies between 0 (complete separation) to 1 (complete overlap). The Pianka index is calculated using the formula as:

${O_{jk}} = \displaystyle{{\mathop \sum \nolimits_{i = 1}^n {p_{ij}}{p_{ik}}} \over {\sqrt {\mathop \sum \nolimits_{n = 1}^n p_{ij}^2\mathop \sum \nolimits_{i = 1}^n p_{ik}^2} }}$

where p_ij_ (p_ik_) is the proportion of food category i recorded in the diet of the species j (or k).

## Results

### Spatial overlap

**Figure 3:**
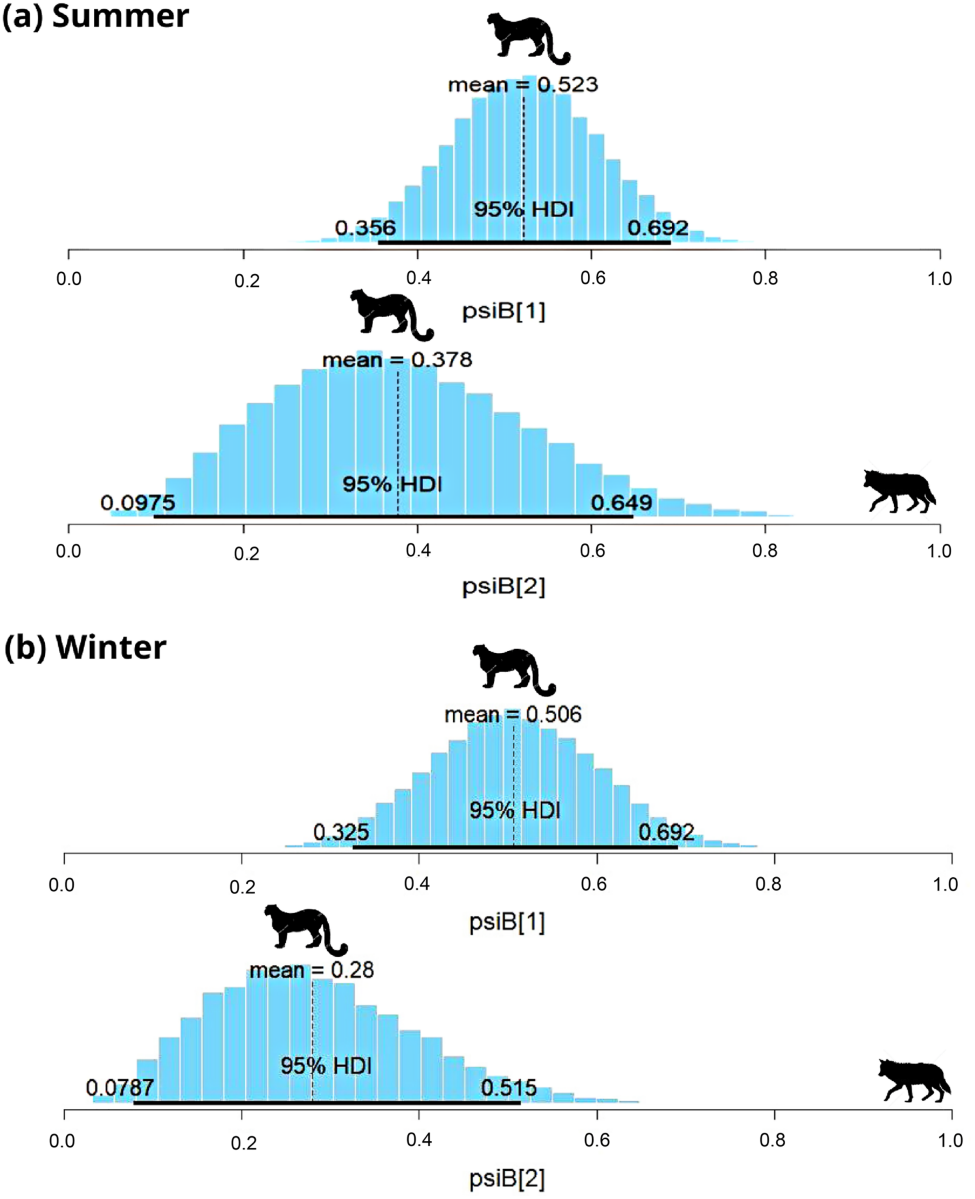
Results of two species occupancy (woolly wolf and snow leopard) for (A) summer and (B) winter. psiB[1] is the occupancy of snow leopard if wolf is absent and psiB[2] represent occupancy of snow leopard in the presence of wolf.

In the Trans-Himalayan region, the snow leopard was captured on 84 occasions and the woolly wolf was captured on 22 occasions in summer. Models with habitat variables (slope, ruggedness, elevation, vegetation index) did not converge; therefore, only the interaction model was used to understand the relationship. The Species Interaction Factor (SIF) for woolly wolf and snow leopard was 0.77 (HDI: 0.2 to 1.3) (Fig. 3A). Since this is not statistically different from one, the model suggests that both species occur independently. The occupancy of the snow leopard changed only slightly in the presence of the woolly wolf; occupancy (* $\psi$*) changed from 0.52 ± 0.08 to 0.37 ± 0.1 (Fig. 3A). During the winter, the snow leopard was captured 57 times and the woolly wolf 48 times. The SIF for the woolly wolf and snow leopard was 0.64 (HDI: 0.19–1.09), again not statistically different from one (Fig. 3B). The model suggests that species occur independently and do not impact the distribution of each other. The presence of snow leopard’s occupancy ( $\psi$) changed slightly from 0.51 ± 0.09 to 0.21 ± 0.12 in the presence of woolly wolf (Fig. 3B).

**Figure 4:**
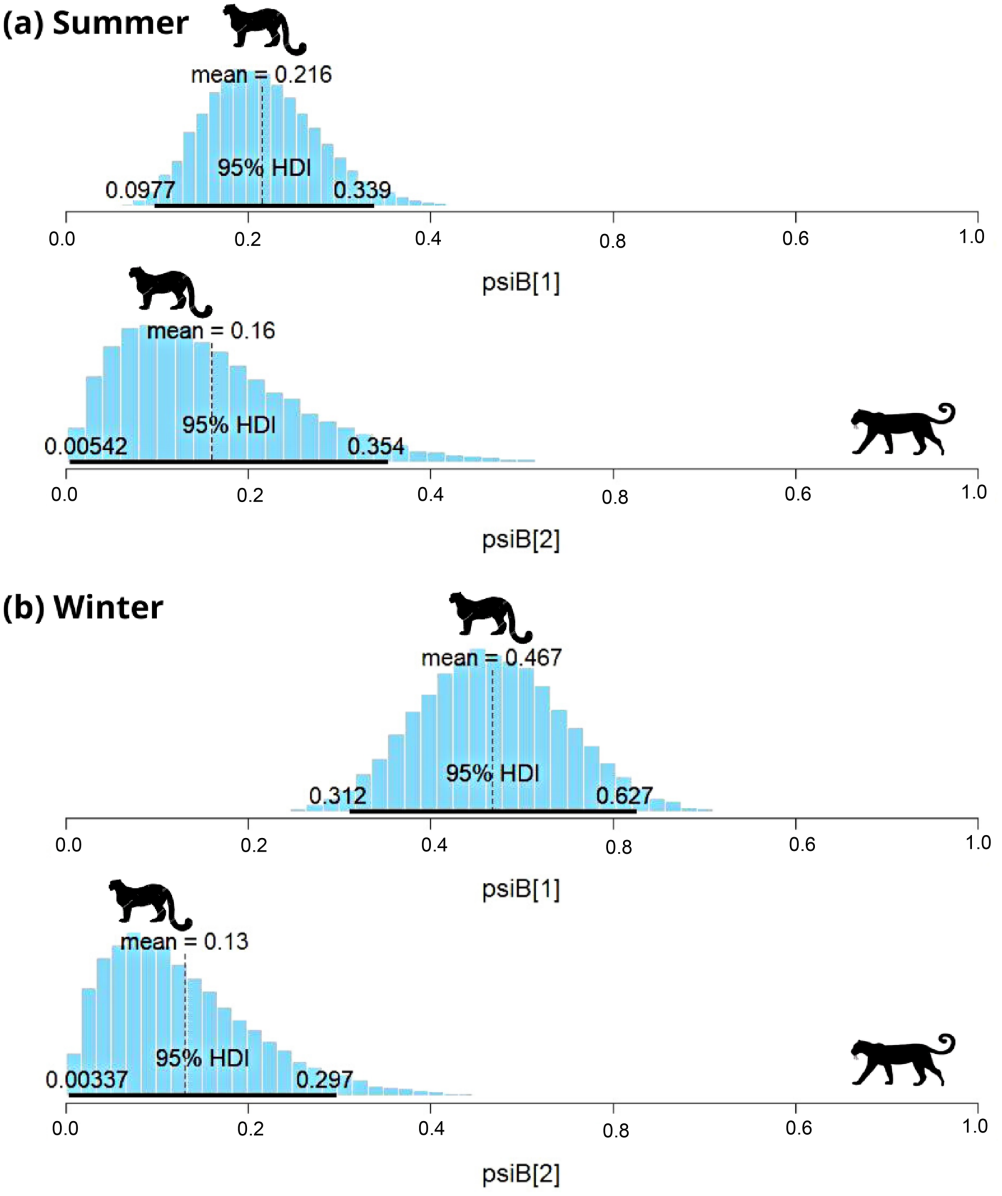
Results of two species occupancy (common leopard and snow leopard) for (A) summer and (B) winter. psiB[1] is the occupancy of snow leopard if common leopard is absent and psiB[2] represent occupancy of snow leopard in the presence of common leopard.

In subalpine and alpine regions, the snow leopard was captured on 29 occasions, and common leopard was captured on 33 occasions in summer. In winter, the snow leopard was captured on 57 occasions, and the common leopard was captured on 41 occasions. Models with habitat variable failed to converge, therefore only the interaction model was used to understand the relationship. During summer, SIF for common leopard and snow leopard was 0.78 (HDI: 0.03 to 1.65) (Fig. 4A). The interaction factor was not statistically different from one, suggesting that both species occur independently. In the presence of common leopard, the occupancy ( $\psi$) of snow leopard showed minor change, from 0.22 ± 0.06 to 0.15 ± 0.1. In contrast to summer, SIF for common leopard and snow leopard (0.3; HDI: 0.02–0.75) was found significant in winter (Fig. 4B). The model suggests that individuals of these species occurred together in this area less than expected. In the presence of common leopard, snow leopard occupancy ( $\psi$) changed from 0.47 ± 0.08 to 0.13 ± 0.08 in winters (Fig. 4B).

### Diet

For wolves, the undigested matter was present in the following decreasing order: hair (100%, present in all scats), bone (81.25%), hoof (18.75%), claws (3.12%), feathers (9.75%), remains of grass (6.25%) and eggshells (6.25%). The mean percentage frequency of occurrence and relative biomass was highest for bharal, followed by livestock, marmot, birds, and small prey (Table 1).

**Table 1:** Content of 32 woolly wolf and 15 snow leopard scat from trans Himalayan habitat of Upper Bhagirathi basin, Uttarakhand, India.

Prey species	Snow leopard		Woolly wolf	
	% Frequency of occurrence (95% CI)	% Relative biomass	% Frequency of occurrence (95% CI)	% Relative biomass
Bharal *Pseudois nayaur*	40 [20–60]	44.93	59 [42–77]	66.34
Livestock	53 [26–80]	42.38	28 [12–43]	19.99
Marmot *Marmota himalayana*	10 [3–30]	7.85	15 [3–25]	7.67
Bird	6 [0–20]	2.44	13 [3–25]	3.9
Small prey	6 [0–20]	2.4	6 [0–15]	2.1

**Note:**

The table describes the mean percentage frequency of prey items (with 95% confidence intervals (CI) from bootstrapping), and the estimated % relative biomass of the prey consumed.

A total of 54 scats were analyzed for snow leopard collected from greater Himalayan (39) and Trans-Himalayan regions (15). Only 11.11% of scats had more than one prey species. The undigested matter was present in the following decreasing order: hair (100%, present in all scats), bone (59.2%), remains of grass (37.03%), hoof (11.11%), claws (7.4%), feathers (1.8%) and teeth (1.85%). In the greater Himalayan habitat, the frequency of occurrence of bharal and livestock was similar, followed by small rodents, musk deer, Himalayan tahr, Himalayan langur, and birds (Table 2). In Trans-Himalaya, snow leopard scat analysis indicated a much narrower range of prey: bharal followed by livestock, marmots, and small prey (rodents and birds) (Table 1).

**Table 2:** Content of 39 snow leopard and 24 common leopard scats from greater Himalayan habitat of Upper Bhagirathi basin, Uttarakhand, India. The table describes the mean percentage frequency of prey items with 95% confidence intervals (CI) from bootstrapping, estimated % relative biomass of the prey consumed.

Prey species	Snow leopard		Common leopard	
	% Frequency of occurrence (95% CI)	% Relative biomass	% Frequency of occurrence (95% CI)	% Relative biomass
Bharal *Pseudois nayaur*	25 [13–4]	32.05	4.17 [0–12]	6.10
Livestock	25 [12–41]	23.76	39.58 [20–59]	42.17
Bird	2 [0–7]	0.96	4.17 [0–12]	1.81
Small prey	23 [10–35]	10.36	29.17 [9–41]	11.12
Musk deer *Moschus leucogaster*	20 [7–33]	17.32	4.17 [0–12]	4.18
Himalayan Tahr *Hemitragus jemlahicus*	7 [0–17]	13.08	–	–
Himalayan langur *Semnopithecus ajax*	5 [0–7]	2.48	–	–
Goral *Naemorhedus goral*	–	–	6.25 [0–15]	8.08
Serow *Capricornis sumatraensis thar*	–	–	4.17 [0–12]	6.61
Sambar *Rusa unicolor*	–	–	12.5 [0–25]	19.93
Unknown	1	–	–	–

In common leopard, only two scats out of 24 (4.8%) had more than one prey species. The undigested matter was present in the following decreasing order: hair (100%, present in all scats), bone (75%), remains of grass (20.8), hoof (4.16%), claws (8.33%), and teeth (4.16%). Rodents were the most frequently found prey item, followed by livestock (Table 2). Wild ungulates in the landscape were also found in leopard’s diet (Table 2). The mean relative biomass was highest for livestock (42.17%), followed by sambar and rodents. Wild ungulates contribute 44.90% of biomass to the leopard’s diet.

Pianka Index for dietary niche overlap between snow leopard and woolly wolf in the Trans-Himalayan valley was 0.8, and 0.75 for the snow leopard and common leopard in the greater Himalayan region.

### Activity pattern

**Figure 5:**
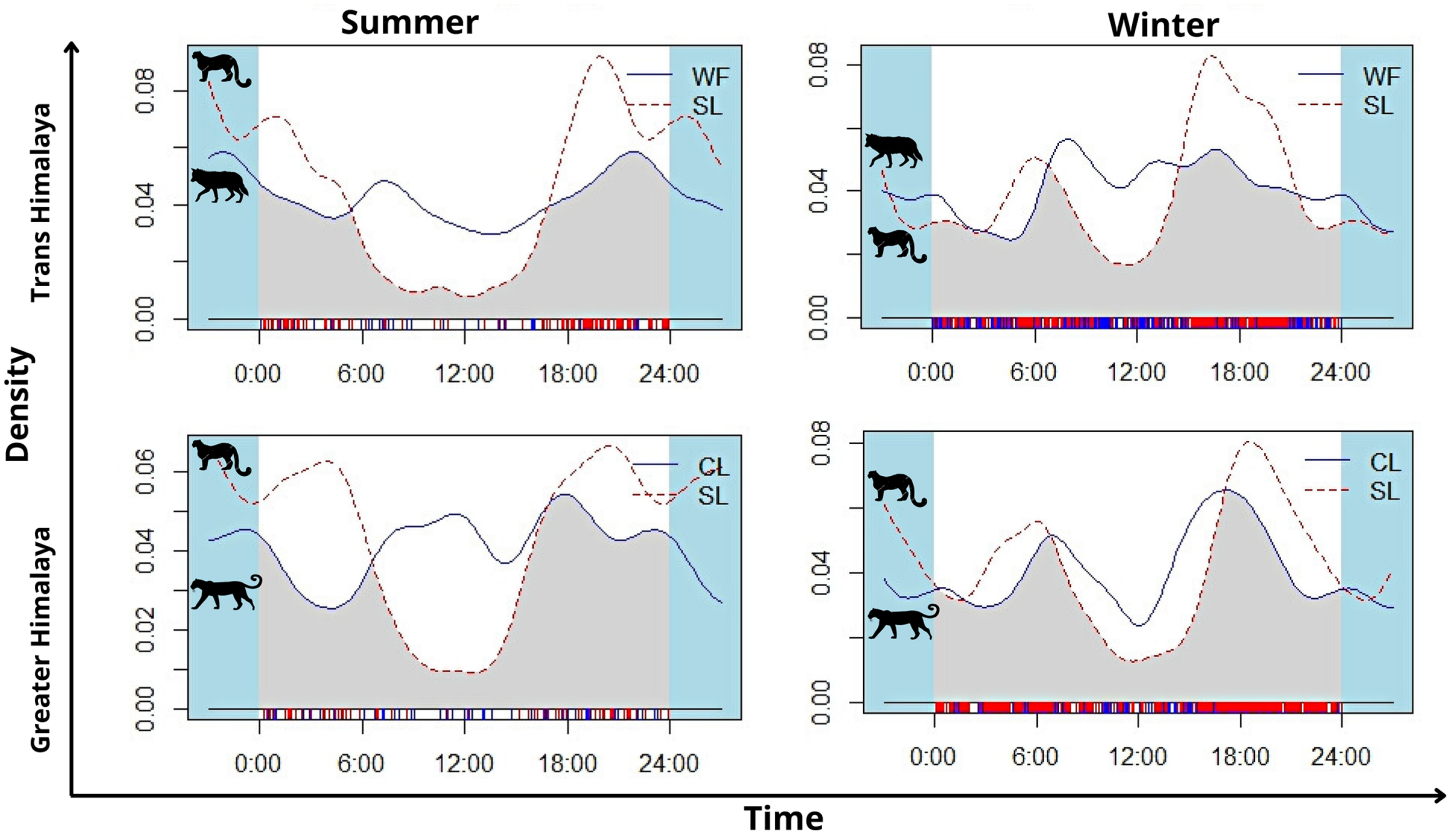
The activity pattern and overlap (grey area) of woolly wolf (WF) and snow leopard (SL) and common leopard (CL) and snow leopard (SL) in the Upper Bhagirathi Basin, Western Himalaya, India.

In summer, snow leopard showed bimodal activity patterns in both the greater Himalayan (*N* = 62) and Trans-Himalayan region (*N* = 104) (Fig. 5). In winters, a similar bimodal activity pattern is observed from greater (*N* = 466) and Trans-Himalayan region (*N* = 258) (Fig. 5). In contrast, the common leopard (*N* = 59) did not show a clear activity pattern (cathemeral activity pattern) in summer (Fig. 5). In winter (*N* = 206), the common leopard showed a bimodal activity pattern with high activity during dawn and dusk (Fig. 5). The woolly wolves were active throughout the day but showed peak activity during early morning and mid-night in summers (*N* = 48) (Fig. 5). In winters (*N* = 391), wolves showed a cathemeral activity pattern (Fig. 5). The activity overlap between snow leopard and common leopard showed 74% overlap in summer and 83% in winters (Fig. 5). In the case of wolf and snow leopard, the temporal overlap was 74% in summer and 80% in winter (Fig. 5).

## Discussion

Activity patterns, space use, and diet are the most commonly used ecological niche axes to determine the extent of interspecific interaction among species (Rayan & Linkie, 2016). Environmental changes can affect the distribution of resources and thus determine the intensity of interactions, yet its role is rarely acknowledged in conservation practices. Carnivores are particularly vulnerable to such changes. Because of their apex position in the food chain, carnivores are likely to be more vulnerable to changes in resource availability and species interactions caused by anthropogenic impacts or climate change (Rubidge et al., 2011; Ockendon et al., 2014). By examining the seasonal spatial and temporal interactions of apex predators in a high-altitude landscape, the study provided insights into the effect of environmental change on species interactions.

### Snow leopard and woolly wolf interactions

Our hypothesis on the negative effect of wolves on snow leopard space use was not supported on spatial scale. Snow leopard and wolf were found to co-occur independently. Species with similar ecological needs can coexist using different habitat features (Gause, 1934; MacArthur, 1958). We could not directly account for the effect of topographic features on co-occurrence patterns, and used the habitat use information (Pal et al., 2022a, 2022b) to understand habitat preferences and plausible mechanisms of co-occurrence. An assessment of habitat use over 4 years in the Upper Bhagirathi basin have shown that snow leopard and woolly wolves differ in their preference for distinct topographic features. Snow leopard prefer steep terrain (Pal et al., 2022a), a pattern well documented in other studies (Jackson & Ahlborn, 1989; Chundawat, 1990; Jackson, 1996). Rugged areas such as cliffs, broken terrain, and overhanging rock faces are considered important for marking, resting, hunting, and escape shelter for the snow leopard, and the cursorial hunting mode of wolves is best suited to less rugged terrain (Chetri, Odden & Wegge, 2017; Pal et al., 2022b; Kachel, Karimov & Wirsing, 2022). Both the predators may use different habitats to maximise the efficacy of their contrasting hunting modes and access to prey, or snow leopard may use marginal habitats to avoid interference interactions with wolves (Lovari et al., 2013a; Kachel, Karimov & Wirsing, 2022). The preliminary analysis of diet overlap indicated a high overlap between woolly wolves and snow leopard. Such overlap is likely due to the absence of diverse prey species in the region. Both snow leopard and woolly wolves in the Trans-Himalayan region are dependent on bharal, the only wild ungulate found in the area. Even though livestock is available for four months (June to September), it significantly contributes to the diets of wolves and snow leopard.

Temporal partitioning is common in carnivore species pairs with distinct dominance hierarchies (Palomares & Caro, 1999). However, such patterns were lacking in the case of wolves and snow leopard. During the summer, both species showed similar crepuscular activity patterns, and both demonstrated minimal activity during peak human activity hours (see Pal et al., 2021a; Pal et al., 2022b). Snow leopards had a similar crepuscular activity pattern across the habitat types in both seasons. Radio collar investigations have also revealed crepuscular activity in snow leopards (Jackson & Ahlborn, 1989; Johansson, 2017). On the other hand, the wolf does not appear to have a distinct bimodal activity pattern in winter. Human activity is relatively low in Nelang during the winter, which may have encouraged wolves to shift their activity to the daytime (Pal et al., 2022b). Conversely, snow leopard continued to follow the crepuscular activity pattern and showed lesser overlap with wolves in winter.

### Snow leopard and common leopard interactions

Contrary to our expectation, we found snow leopard and common leopard temporarily and spatially separated during the summer season. However, in the winter, the common leopard negatively influenced the space use of the snow leopard. Common leopards in the Upper Bhagirathi basin were photo captured up to 3,500 m (subalpine habitat). Snow leopards were photographed mainly in alpine habitats, but also in subalpine and temperate habitats of the basin (2,700–3,500 m). In the summer, snow leopards may move to higher elevations with less snow and occur independently of common leopard presence. During winter, seasonal movement is widely documented in prey species of snow leopard (Schaller, 1998; Anwar & Minhas, 2008; Dendup & Lham, 2018; Pal et al., 2021a, 2021b) which are known to shift to lower elevations due to excessive snowfall. Such behavior is also expected in snow leopards (following prey species) in the winter season, where chances of encounters and competition for space increase with common leopard. In the greater Himalayan region, with a diverse range of species available, snow leopards and common leopard feed on various wild ungulates. Bharal and musk deer are the wild prey found common in the diet of both the felids, but the common leopard’s diet was more diverse than snow leopard. High dietary overlap between snow leopard and common leopard is primarily due to the high proportion of livestock in the diet of both species. In the absence of livestock during winter, competition among predators for prey is likely to increase.

In the greater Himalaya, snow leopard showed the crepuscular activity pattern (similar to the Trans-Himalayan habitat) in both seasons. It is known that under certain circumstances carnivores adjust their activities to the availability of prey (Jenny & Zuberbuhler, 2005; Salvatori et al., 2021). The common leopard showed a similar pattern in summer, showing diurnal activity which corresponds closely to livestock grazing (see Pal et al., 2021a). In winter, the common leopard showed a bimodal activity pattern similar to that of the snow leopard, with a high overlap in activity patterns.

### Drivers of intraguild interactions

According to the competitive exclusion principle (Gause, 1934), sympatric predators should partition dietary, spatial, and/or temporal niche dimensions to achieve some level of resource independence that allows stable coexistence (Chesson, 2000). Similar dietary needs and activity patterns between the wolf and snow leopard expect considerable competition between these species. However, habitat heterogeneity and differences in habitat use are likely to limit the competition between snow leopard and wolves, facilitating their co-occurrence in the region. This is consistent with the results of Salvatori et al. (2021) from Mongolia, which found a similar co-occurrence pattern of snow leopard and wolf. A recent study from the Pamir mountains in Tajikistan, conducted at finer scale, found overlapping temporal and dietary niches, but a differential use of habitat type between wolves and snow leopard (Kachel, Karimov & Wirsing, 2022). In the case of snow leopard and common leopard, the negative spatial interaction was limited to scarce resource season, when livestock is absent, and space available is limited due to heavy snow. A study on the interaction between two leopard species in the Sagarmatha National Park showed a high overlap in prey categories and sizes but habitat separation between these two felids (Lovari, Ventimiglia & Minder, 2013b). We believe that, in our case, limited prey resources (lack of livestock) and similar activity patterns in winters may result in intense competition, causing these species to avoid each other on a spatial scale. Melting snow in the higher elevations in summers opens up rugged and alpine habitats and ridgelines for snow leopards and other prey species. Heavy snow in higher elevation locations or the absence of anthropogenic influences in winter cause prey species to shift/expand to lower elevation places. Such a shift in prey species is likely to promote snow leopard utilization of low elevation habitat, increasing spatial overlap between the two leopard species.

Body size appears to be the most crucial determinant of aggressive interaction in carnivores (Donadio & Buskirk, 2006). In the case of large and small differences, intense competition is less likely to occur, whereas, at intermediate differences, intraguild killings are more common (Donadio & Buskirk, 2006). The degree of competition is also influenced by taxonomic relatedness. Species within a family tend to have high similarities in life-history traits than species from other families (Gittleman, 1985; Van Valkenburgh, 1991). As a result, competition is expected to be more lethal within than among families (Donadio & Buskirk, 2006). Furthermore, in seasonal environments, the majority of agonistic encounters between carnivore species occur when food is scarce (Palomares & Caro, 1999). We aimed to understand the interactions of large predators under varying conditions by examining seasonal habitat conditions and prey availability. Wolf and snow leopard, carnivores belonging to different families, appear to co-occur independently in summer and winter habitat conditions. Even though they both rely on the same prey species in the area, the heterogeneous habitat conditions and preference for different terrain types promote their co-existence. On the contrary, snow and common leopard, carnivores from the same family, showed contrasting patterns in distinct ecological settings. Resource-rich seasons allow for minimal competition in terms of prey and accessible area; however, when resources are scarce, both species appear to compete for space. In the latter circumstances, a species with dominance and a more adaptive, broader dietary and spatial niche, such as the common leopard in our case, is likely to predominate.

It is likely that we may not have detected all possible finer-scale intra-guild carnivore adaptations. Research with more scat samples can help understand the full spectrum of diet, seasonality in prey dependence, and competition for prey. The effect of various topographic features, such as topographic complexity, elevation, and prey availability, could be conclusively quantified with a larger dataset. Further research is needed to understand the other factors that can influence intraguild relations, such as the interactive effect of density thresholds, anthropogenic settings, and the availability and spatial distribution of feeding resources (Palomares et al., 1996; Karanth et al., 2017; Sivy et al., 2017). Investigating the interactions of snow leopards with co-predators in various abiotic and biotic settings can help to provide such understanding.

## Conclusion

Interspecies interactions among carnivores impact predator fitness, distribution, and overall community structure. Given the ongoing worldwide extirpation of carnivore guilds and prey communities (Wolf & Ripple, 2016), as well as the role of anthropogenic disturbance as a mediator of intraguild interactions (Manlick & Pauli, 2020; Sévêque et al., 2022), understanding niche partitioning patterns is critical for conservation planning (Lahkar et al., 2021). Our study showed that the nature and strength of species interactions are sensitive to varying environmental conditions and resource shifts. Since intra-guild sympatry among the species can be facilitated by habitat availability and heterogeneity, delineating such habitat in conservation planning is essential. Furthermore, adequate prey diversity and availability are crucial for the stable coexistence of the carnivore guild. These considerations are likely to become more important as intact habitats become scarce, prey species decline (Powers & Jetz, 2019), and large carnivores are forced into smaller, more fragmented areas (Wolf & Ripple, 2017). Conservation of entire carnivore guilds is critical for maintaining the ecological stability of any habitat; thus, preserving large enough areas with diverse habitats, as well as characterization of species requirements along different niche dimensions, is crucial. It is especially crucial in the high-altitude, heterogeneous Himalayan landscape, where the availability of resources is highly seasonal and vulnerable to human pressure.

Additionally, climate change is expected to reshuffle the ecological communities, altering resource availability, changing species interactions, and generating novel interactions (Alexander et al., 2016). Documenting the occurrence of niche partitioning and determining the mechanistic drivers and interspecific relationships of such partitioning is essential to understanding the community ecology to forecast the direct and indirect repercussions of environmental change (Alexander et al., 2016). Recent climate-based habitat models for the snow leopard range suggest a projected loss of suitable habitat in the southern range of the species, owing primarily to an upward treeline shift (Forrest et al., 2012; Lovari et al., 2013a; Aryal et al., 2016). In such scenarios, the interaction of snow leopards with wolves and common leopards is expected to increase. Wolves and snow leopards may coexist in a topographically diverse environment if sufficient prey is available. On the other hand, a shift in tree line habitat may result in severe competitive interactions between common leopard and snow leopard, which might prove detrimental to snow leopard. Our initial results indicate that the snow leopard’s future distribution faces more challenges than habitat loss due to tree line shifts.

## Supplemental Information

10.7717/peerj.14277/supp-1 Supplemental Information 1 Raw data of the common leopard and snow leopard for summer for two species occupancy analysis in R software using the package R2Jags.

10.7717/peerj.14277/supp-2 Supplemental Information 2 Raw data of the common leopard and snow leopard for winter for two species occupancy analysis in R software using the package R2Jags.

10.7717/peerj.14277/supp-3 Supplemental Information 3 Raw data of the woolly wolf and snow leopard for summer for two species occupancy analysis in R software using the package R2Jags.

10.7717/peerj.14277/supp-4 Supplemental Information 4 Raw data of the woolly wolf and snow leopard for winter for two species occupancy analysis in R software using the package R2Jags.

10.7717/peerj.14277/supp-5 Supplemental Information 5 Raw seasonal (summer and winter) data of snow leopard (greater and trans-Himalaya), common leopard, and woolly wolf for activity pattern and temporal overlap analysis in R software using the Overlap package.

10.7717/peerj.14277/supp-6 Supplemental Information 6 Raw data on diet of snow leopard (greater and trans-Himalaya and greater Himalaya), common leopard, and woolly wolf.

## Additional Information and Declarations

### Field Study Permissions

The following information was supplied relating to field study approvals (*i.e*., approving body and any reference numbers): Field sampling was approved by Uttarakhand Forest Department (approval numbers: 836/5-6, 702/5-6, 117/5-6).

### Data Availability

The following information was supplied regarding data availability: The raw data are available in the Supplemental Files.
